# The experience of disproportionate supplies of COVID-19 vaccines to Africa: the need for Africa to decolonize its public health response

**DOI:** 10.11604/pamj.2023.44.54.37737

**Published:** 2023-01-27

**Authors:** Adeponle Olayode Adeoye, John Lola Okunola, Sunday Olutayo Fakunle

**Affiliations:** 1 Department of Sociology, Redeemer’s University, Ede, Osun State, Nigeria

**Keywords:** COVID-19, vaccine, Africa, inequity

## Abstract

The recent outbreak of COVID-19 resulted in high fatality rates globally and huge socio-economic consequences. Vaccine development was considered as the most effective and fastest means to reduce the risks associated with the disease. All hands were on deck for effective vaccine development which resulted in several countries including African countries participating in the vaccine solidarity trial. At the heat of the pandemic in 2021, Africa with a population of 1.4 billion had 134.5 million supplies of COVID-19 vaccines. The United States in contrast with a population of 332 million had over 375 million doses of the vaccine. This was one of the factors that hindered most African countries from meeting up with the World Health Organization (WHO) target of 40% fully vaccinated individuals by December 2021. Even in some African countries where there were early and good supplies, there was vaccine wastage due to near to expire vaccines supplied to such countries which resulted in misconception and rejection of the vaccines among the public. This paper discussed the politics of COVID-19 vaccine production and distribution with its effects on Africa using documentary analysis. The paper also suggests strategic bold steps needed to be taken by African countries to decolonize public health response within the continent in preparation for future pandemics.

## Essay

Vaccine development is one of the greatest milestones in health science that has contributed to the sustenance and biological adaptation of the human race to the environment in spite of the existing health threats [1,2]. Vaccine production is a scientific activity that involves developing an efficacious therapy that can strengthen the immune system to fight or resist a disease [3-5]. Vaccine has no doubt increased life expectancy, reduced mortality, and enhanced economic growth [6]. Vaccines can be prophylactic or therapeutic [7]. Prophylactic offers protection against diseases yet to occur or reduce the effect when it eventually occurs while therapeutic vaccines cure or fight diseases that have already occurred [8]. According to the World Health Organization, vaccines have been developed and licensed for twenty-five different preventable diseases [9], although this might have increased due to emerging and re-emerging diseases that have triggered vaccine research and development.

Vaccine production is a challenging task with different phases which is difficult to execute but necessary to produce a safe and effective vaccine [10]. Currently, there are few vaccine manufacturers in the world because of the daunting nature of the industry which involves huge expenses and low chances of success rate [1]. Vaccine production has 94% chance of failure [11]. High-income countries have been responsible for vaccine production while Africa with the highest incidence of deaths caused by infectious diseases has no capacity to produce vaccines [6]. Africa imports 99% of its vaccines [12]. As a result of the over-centralization of vaccine production in the west, over 9 million children in Africa are hindered from receiving the full dose of diphtheria, tetanus, and pertussis immunization due to the scarcity of these vaccines in some parts of Africa [12].

As of June 2022, only 14.7% of people in low-income countries and 53.8% in middle-income countries have been vaccinated against COVID-19 as compared to 74.8% and 75.9% in high-income and upper-middle-income countries [13]. Although the widespread information was that the impact of COVID-19 was not severe in Africa but recent serological studies have reported a high presence of COVID-19 antibodies in Africa and that 67% of the African population might have been infected with COVID-19 [14].

**The politics of COVID-19 vaccine production:** science is often used to advance political, economic, and cultural agenda [15]. This is clear in vaccine production as the process is often influenced with politics and economic aspirations that compromise access and acceptance of vaccines [16]. Developed nations have monopolized vaccine production, increasing global health inequality and the dependency of developing nations [17]. The politicization of COVID-19 vaccines was evident through the stiff resistance of COVID-19 vaccine producers to the temporary ‘Intellectual Property Waiver´ initiated to enhance rapid local production and access to COVID-19 vaccines in low- and middle-income countries [18].

The resistance was on the ground that developing countries like Africa lack the scientific and technical capacity to produce vaccines in large quantities and such a policy would hamper access to quality vaccines [17,18]. However, the World Health Organization acknowledged in the year 2021 that there are about 80 sterile injectable facilities in Africa which is a great potential for vaccine production [19]. Instead of strengthening Africa´s capacity for production by training and repurposing the existing infrastructure, the developed countries through the vaccine producers frustrated every effort to ensure that vaccine production in developing countries does not come into fruition [17].

The temporary “Intellectual Property Waiver” was merely an eye service by the west. European countries went to the extent of making the exportation of raw materials critical for the production of the vaccine difficult for some countries such as India even after the announcement of the waiver [20]. Instead, high-income countries resorted to vaccine charity and donation for low-and middle-income countries. Strengthening the capacity for local production of COVID-19 vaccines would have made a long-term positive impact on the healthcare systems in low- and middle-income countries and enhance proactive response to present and future epidemics in these countries.

**COVID-19 vaccine inequity in Africa:** at the heat of the pandemic and after approval had been given for the use of COVID-19 vaccines in late 2020, African had supplies of about 134.5 million of COVID-19 for a population of about 1.4 billion people as at August 2021 [21]. The United States in contrast had over 375 million COVID-19 vaccines for a population of about 332 million people [21]. Self-interests characterized the distribution of COVID-19 vaccines with the Global North hoarding and stockpiling large quantities of vaccines with disproportionate supplies to Global South [22]. It must be noted that some high-income countries were already stockpiling for boosters when some low- and middle-income countries were still struggling to administer the first dose for their citizens.

COVID-19 Vaccines Global Access (COVAX) was set up to ensure justice in the distribution of the vaccines globally but before the vaccine was given formal authorization for use, the Global North had engaged in vaccine nationalism by pre-purchasing all COVID-19 vaccines that showed the most promising results [13]. For instance, the United Kingdom signed an agreement with pharmaceutical companies such as BioNtech/Pfizer, AstraZeneca, Valneva and GSK/Sanofi [23] in order to secure a stock of the vaccine for its population even before the vaccine was given formal approval for use. The US, France, Germany and Italy also signed a direct deal with BioNtech/Pfizer and Moderna prior to the vaccine approval for use [24]. After the vaccines had been formally approved for use, Africa was unable to secure early fair pricing deals for some vaccines due to bilateral agreements of high-income countries with the vaccine producers which increased the price of the vaccines [25]. After the western world had fully vaccinated their citizens, most of them then engaged in donation of near to expire stockpiled vaccines which resulted in vaccine wastage and hesitancy, increasing the risk of Africans from suffering from the severity of new variants of COVID-19, delayed recovery of African fragile economy and inability of most African nations to achieve WHO target of 40% rate of fully vaccinated individuals by December 2021 [21].

**Decolonizing public health response in Africa: the way forward:** although the fatalities associated with COVID-19 was global, which resulted in stiff competition for vaccine, putting low resource countries at the risk of not getting adequate supplies of vaccines needed to win the battle of the virus over its citizens and economy. The inequity in the vaccines supplied to Africa at the early stage of the vaccine approval, and heat of the pandemic has necessitated the need for African countries to reduce their dependency on high-income countries for intervention during epidemic/pandemic outbreak. COVID-19 has proven the adage “every man for himself” especially during pandemic outbreak. The citizens of the nations where vaccines are developed are the first priority before any other nation.

It is important that Africa take a bold step towards decolonizing public health response in preparation for future pandemics and in response to other endemic diseases within the continent. Africa must wake up from its slumber as regards vaccine production. Vaccines remain the most effective response to disease containment and eradication which is also quite expensive and also involve social costs.

Vaccine production and other therapeutics begin with significant investment in research. The research is complex and divided into steps which include; early research phase, patent application, pre-clinical testing phase, and the three stages of the clinical trials [20]. Each of these phases requires huge financial investment. Most developed countries are leading in vaccine production because of their large investment in vaccine research [26]. The cost of developing a single infectious disease vaccine from pre-clinical trials to the end of phase 2 of clinical trial is between 2.8-3.7 billion dollars [27]. Africa must be ready to invest robustly in research in order to develop and produce vaccines as part of the steps to decolonize public health response within the continent.

Vaccine production is also dependent on the availability of raw materials and professionals with technical know-how [27]. The raw materials for vaccine production are often competitive which may interrupt its cost and supply [26]. The production process requires a strong scientific educational system, advanced technology, production and storage components which are very expensive, and a strong knowledge of quality control systems [26]. Africa must invest adequately in its educational system to produce individuals with the technical know-how in vaccine production, as well as its public health infrastructure to ensure that the rigour involved in vaccine production is not compromised and safe and effective vaccines are produced.

The social cost has to do with individuals´ willingness to participate in clinical trials. The efficacy and safety of vaccines must be experimented on diverse populations before they can be approved for wide use. It is important to mention that clinical trial is the most delicate and risky part of vaccine research and development. In sub-Saharan Africa, there is often the hesitancy and misperception of clinical trials due to the historical cost of this practice to this part of the continent and cultural issues. For example, the Pfizer Trovan trial in Kano, Nigeria left a long-lasting negative memory of clinical trials in sub-Saharan Africa. In 1996, a team of scientist from Pfizer Pharmaceutical Company in New York came to Nigeria to experiment the efficacy of an anti-biotics called Trovan against meningitis- an epidemic that raged in Northern Nigeria. The trial involved 200 children and resulted in the death of 11 out of the children while some were diagnosed of liver failure and some became paralyzed [28].

Cultural issues in sub-Saharan Africa is another factor that may affect vaccine trial. In a drug clinical trial of HIV/AIDS conducted in Malawi, there was a report that medical staff were attacked during the trial [29]. The locals perceived them to be vampires because the trials involved drawing blood from participants to determine the efficacy of the drug. Due to these reasons, less than 10% of clinical trials are conducted in sub-Saharan Africa [29]. Another study also observed that blood is sacred in most part of African traditional societies as there is the belief that one may become ill after blood has been collected [30]. Therefore, collecting blood samples to test the efficacy of vaccines may be a problem. Therefore, it is important that Africa must strategically address the existing cultural issues associated with clinical trials in Africa and ethical issues associated with vaccine trials must be addressed. The risk associated with vaccine trials must be well communicated to volunteers appropriately and their consent must be appropriately sought without coercion before proceeding with such trials.

**Conclusion:** the inequity in the distribution of COVID-19 vaccines to Africa at the heat of the pandemic highlights the need for Africa to reflect on its public healthcare system and reposition it. The unequal vaccine distribution to Africa no doubt predisposed the continent to the risk of experiencing the severity of new variants and further perpetuated African fragile economy from early recovery. It is therefore, necessary for Africa to decolonize its public health response through strategic repositioning of its public health care system by adequately investing in research, revamping the educational structure by addressing all the weaknesses, development of public healthcare infrastructure, and human capacity development.
